# Gene expression profiling of 49 human tumor xenografts from *in vitro* culture through multiple *in vivo* passages - strategies for data mining in support of therapeutic studies

**DOI:** 10.1186/1471-2164-15-393

**Published:** 2014-05-22

**Authors:** Melinda G Hollingshead, Luke H Stockwin, Sergio Y Alcoser, Dianne L Newton, Benjamin C Orsburn, Carrie A Bonomi, Suzanne D Borgel, Raymond Divelbiss, Kelly M Dougherty, Elizabeth J Hager, Susan L Holbeck, Gurmeet Kaur, David J Kimmel, Mark W Kunkel, Angelena Millione, Michael E Mullendore, Howard Stotler, Jerry Collins

**Affiliations:** Biological Testing Branch, National Cancer Institute at Frederick, 1050 Boyles Street, Building 1043, Room 11, Frederick, MD 21702 USA; Biological Testing Branch, Developmental Therapeutics Program, Leidos Biomedical Research, Inc., Frederick National Laboratory for Cancer Research, Frederick, MD 21702 USA; Biological Testing Branch, Developmental Therapeutics Program, National Cancer Institute at Frederick, Frederick, MD 21702 USA; Thermo Fisher, 134 Yorkshire Boulevard, Indianapolis, IN USA; Information Technology Branch, Developmental Therapeutics Program, Division of Cancer Treatment and Diagnosis, NCI, Bethesda, MD 20892 USA; Molecular Pharmacology Branch, Developmental Therapeutics Program, National Cancer Institute at Frederick, Frederick, MD 21702 USA; Developmental Therapeutics Program, Division of Cancer Treatment and Diagnosis, NCI, Bethesda, MD 20892 USA

**Keywords:** Xenograft models, Affymetrix HG-U133 Plus 2.0 array, cDNA microarray, NCI-60 cell line screen, Transcriptomic stability, Transcriptomic expression, *in vitro* to *in vivo* transition

## Abstract

**Background:**

Development of cancer therapeutics partially depends upon selection of appropriate animal models. Therefore, improvements to model selection are beneficial.

**Results:**

Forty-nine human tumor xenografts at *in vivo* passages 1, 4 and 10 were subjected to cDNA microarray analysis yielding a dataset of 823 Affymetrix HG-U133 Plus 2.0 arrays. To illustrate mining strategies supporting therapeutic studies, transcript expression was determined: 1) relative to other models, 2) with successive *in vivo* passage, and 3) during the *in vitro* to *in vivo* transition. Ranking models according to relative transcript expression *in vivo* has the potential to improve initial model selection. For example, combining p53 tumor expression data with mutational status could guide selection of tumors for therapeutic studies of agents where p53 status purportedly affects efficacy (e.g., MK-1775). The utility of monitoring changes in gene expression with extended in vivo tumor passages was illustrated by focused studies of drug resistance mediators and receptor tyrosine kinases. Noteworthy observations included a significant decline in HCT-15 colon xenograft ABCB1 transporter expression and increased expression of the kinase KIT in A549 with serial passage. These trends predict sensitivity to agents such as paclitaxel (ABCB1 substrate) and imatinib (c-KIT inhibitor) would be altered with extended passage. Given that gene expression results indicated some models undergo profound changes with *in vivo* passage, a general metric of stability was generated so models could be ranked accordingly. Lastly, changes occurring during transition from *in vitro* to *in vivo* growth may have important consequences for therapeutic studies since targets identified *in vitro* could be over- or under-represented when tumor cells adapt to *in vivo* growth. A comprehensive list of mouse transcripts capable of cross-hybridizing with human probe sets on the HG-U133 Plus 2.0 array was generated. Removal of the murine artifacts followed by pairwise analysis of *in vitro* cells with respective passage 1 xenografts and GO analysis illustrates the complex interplay that each model has with the host microenvironment.

**Conclusions:**

This study provides strategies to aid selection of xenograft models for therapeutic studies. These data highlight the dynamic nature of xenograft models and emphasize the importance of maintaining passage consistency throughout experiments.

**Electronic supplementary material:**

The online version of this article (doi: 10.1186/1471-2164-15-393) contains supplementary material, which is available to authorized users.

## Background

Xenograft models remain a cornerstone technology in the development of anti-cancer agents [1]. The ability of immunocompromised rodents to support the growth of human tumors provides an invaluable transition between *in vitro* testing, pre-clinical development and clinical trials. For decades, data from xenograft models has informed development decisions with respect to dosing schedules, pharmacokinetics/pharmacodynamics (PK/PD) and potential toxicities. Yet several challenges remain, including understanding the extent to which well-characterized xenograft models replicate the biology and growth characteristics of patient disease. Furthermore, with the paradigm shift towards agents with specific molecular targets and personalized medicine, a comprehensive molecular profile for xenograft models may be essential for successful *in vivo* evaluation. To this end, considerable resources are being applied to the generation of novel xenograft models combined with molecular profiling of existing models [2].

Within the Developmental Therapeutics Program (DTP) of the National Cancer Institute, the primary *in vitro* assay used to detect potential anti-cancer activity is the NCI-60 cell line screen. To date, this panel of human tumor lines has been used to evaluate almost one hundred thousand pure compounds and approximately fifty thousand natural product extracts. Many of these cell lines will grow as subcutaneous xenografts, thus cell lines sensitive to an agent *in vitro* were often subjected to further analyses in xenografts derived from those cell lines. The NCI-60 panel has been extensively molecularly characterized, with data available for gene expression, DNA variation (mutation and SNPs), protein expression, DNA methylation, microRNA expression and metabolomics (http://dtp.cancer.gov/mtargets/mt_index.html) [3–9]. The COMPARE algorithm (http://dtp.nci.nih.gov/docs/compare/compare.html) allows investigators to correlate NCI-60 drug activity profiles with all other open agents in the database and with molecular characteristics of the cells [10]. While the *in vitro* grown cells have been characterized, the corresponding subcutaneous xenografts had not. However, other studies have succeeded in molecular profiling of other xenografts [2, 11, 12]. These predominantly cDNA and tissue-array based studies lack the potential for retrospective cross-platform analysis that underscores the NCI-60 cell line set.

The MicroXeno Project was initiated to generate genome-wide cDNA microarray data for all subcutaneous xenograft models currently used within the DTP. Comprehensive transcriptomic analysis will help address questions such as: Can expression of molecular target[s] help inform model selection for a given agent or target, or to what extent and by what manner do specific cell line tumors adapt during prolonged growth *in vivo*? The work presented here provides a reference dataset that can be used to confirm the genetic characteristics and stability of models going forward. This ongoing project will ultimately encompass over 100 models, with data from cell lines and successive xenograft passages. The panel includes tumor cell lines from diverse histological origins including leukemia/lymphoma, non-small cell lung, colon, CNS, melanoma, ovarian, renal, prostate, liver, gastric, head and neck, pancreatic, and breast cancer. It also includes, but is not limited to, many of the cell lines in the NCI-60 panel (http://dtp.cancer.gov/docs/misc/common_files/cell_list.html).

Here we describe the procedures and quality control criteria used to derive MicroXeno release 1.0. The study utilizes Affymetrix HG-U133 Plus 2.0 arrays, capable of identifying more that 30,000 human transcripts from >54,000 probe sets [13]. The current release encompasses 49 experiments with each consisting of data from the originating cell lines and the resulting xenografts at passages 1, 4 and 10 (P1, P4, P10). In addition, we detail several approaches to utilize the data to predict possible biological outcomes. We propose that this dataset [and subsequent releases] will improve selection and execution of subcutaneous xenograft experiments during the evaluation of cancer therapeutics. As this study is based primarily on lines from the NCI-60 panel, the potential also exists for integration with other NCI-60 datasets (mutational analysis, protein arrays, etc.). Rapid dissemination of this data will also permit the extramural community to perform meta-analyses in support of their own *in vivo* studies.

## Results and discussion

This study focused on generating pan-genomic cDNA microarray data for diverse xenograft models from the time of initial implantation to the tenth passage using the Affymetrix HG-U133 Plus 2.0 array. Each experiment comprised the originating *in vitro* cell lines [designated P0] along with tumor samples from *in vivo* passages 1, 4 and 10. For each *in vivo* passage, five tumors were harvested. The 49 human xenograft experiments contained within this release led to the generation of 844 arrays. Table 1 details the specific cell lines along with tumor types, histology, host strain and any special growth requirements or irregularities.

**Table 1:** **Tumor cell lines, host mice, protocol variances**

Tumor type^a^	Tumor name	Histology	Mouse strain	Comments
**Breast**	MCF7^b^	Adenocarcinoma	nu/nu NCr	2 samples P0, 4 samples P1
	MDA-MB-231 T	Adenocarcinoma, from mouse	nu/nu NCr	2 samples P0
**Cervical**	HeLa-Luc	Adenocarcinoma	nu/nu NCr	2 samples P0
**CNS**	SF-268^c^	Anaplastic Astrocytoma	NOD.SCID/NCr	2 samples P0, P4 and P10 are mouse
	SF-539^c^	Glioblastoma	NOD.SCID/NCr	2 samples P0, P4 and P10 are mouse
	U251	Glioblastoma	nu/nu NCr	2 samples P0
**Colon**	COLO 205	Adenocarcinoma	nu/nu NCr	
	HCC-2998	Carcinoma	nu/nu NCr	
	HCT-116	Carcinoma	nu/nu NCr	
	HCT-15	Adenocarcinoma	nu/nu NCr	
	HT-29	Adenocarcinoma, GR III	nu/nu NCr	
	KM12	Adenocarcinoma	nu/nu NCr	
	SW-620	Adenocarcinoma	nu/nu NCr	
**Gastric**	GTL 16	Adenocarcinoma	nu/nu NCr	2 samples P0
**Leukemia/**	AS283	AIDs related Burkitts Lymphoma	SCID/NCr	2 samples P0
**Lymphoma**	CA46	B Lymphocyte Burkitts Lymphoma	SCID/NCr	2 samples P0
	CCRF-CEM	Acute Lymphoblastic Leukemia (T-ALL)	SCID/NCr	
	HL-60(TB)	Promyelocytic Leukemia	SCID/NCr	3 samples P5 substituted for P4; 4 P0 samples
	K-562	Chronic Myelogenous Leukemia	SCID/NCr	5 samples P5 substituted for P4, 4 samples P10
	MOLT-4	Acute Lymphoblastic Leukemia	SCID/NCr	
	SR	Large Cell, Immunoblastic	SCID/NCr	
**Liver**	HuH-7	Differentiated Hepatocellular Carcinoma	nu/nu NCr	2 samples P0
**Lung**	A549	Adenocarcinoma	nu/nu NCr	
	A549/Asc-1	Adenocarcinoma	nu/nu NCr	3 samples P4, 2 samples P0
	EKVX	Adenocarcinoma	SCID/NCr	2 samples P0
	HOP-62	Adenocarcinoma	nu/nu NCr	4 samples P1
	HOP-92	Large Cell, undifferentiated	nu/nu NCr	
	NCI-H226	Squamous Carcinoma	nu/nu NCr	2 samples P1
	NCI-H23	Adenocarcinoma, NSCLC	nu/nu NCr	2 samples P0
	NCI-H460	Large Cell Carcinoma	nu/nu NCr	
	NCI-H522	Adenocarcinoma	nu/nu NCr	2 samples P0
**Melanoma**	A375	Metastatic Malignant Melanoma	nu/nu NCr	2 samples P0
	COLO 829	Malignant Melanoma	nu/nu NCr	2 samples P0
	LOX IMVI	Malignant Amelanocytic Melanoma	nu/nu NCr	2 samples P0
	M14	Adenocarcinoma	nu/nu NCr	2 samples P0
	MALME-3 M	Malignant Melanoma	nu/nu NCr	2 samples P0, 4 samples P4
	MDA-MB-435	Adenocarcinoma	nu/nu NCr	2 samples P0, 3 samples P10
	MDA-N^d^	HER2/ERB2 transfectant of MDA-MB-435	nu/nu NCr	2 samples P0
	UACC-62	Malignant Melanoma	nu/nu NCr	
**Ovarian**	CP70	Carcinoma	nu/nu NCr	2 samples P0
	OVCAR-3	Adenocarcinoma	nu/nu NCr	2 samples P0
	OVCAR-5	Carcinoma	nu/nu NCr	2 samples P0
**Pancreatic**	AsPC-1	Adenocarcinoma	nu/nu NCr	2 samples P0
**Prostate**	PC-3	Adenocarcinoma	Male nu/nu NCr	2 samples P0
	PC-3/M^e^	Metastatic PC-3 subline	Male nu/nu NCr	
**Renal**	786-0	Adenocarcinoma	nu/nu NCr	
	CAKI-1	Renal Cell Carcinoma	nu/nu NCr	2 samples P0
	RXF 393	Poorly Differentiated Hypernephroma	nu/nu NCr	
	SN12C	Carcinoma	nu/nu NCr	2 samples P0

^a^Tumor cells derived from the 4th *in vitro* passage were innoculated subcutaneously into mice. Tumors were passaged 10 consecutive times in mice. At passages 1, 4, and 10 (P1, P4, P10, respectively), 5 tumors were harvested, cut into small pieces and flash frozen in liquid nitrogen. Samples were then processed as described in Methods. For comparative purposes, 3 samples of the *in vitro* cultivated cells at passage 4 (P0) were also prepared for microarray analysis.

^b^3 mg/kg Q7D SC Estradiol.

^c^Matrigel 18.1 mg/mL P1 only.

^d^HER2/ERBB2 transfectant of MDA-MB-435.

^e^PC3 subline isolated from liver metastasis in mice.

### Microarray data quality control

**Figure 1:**
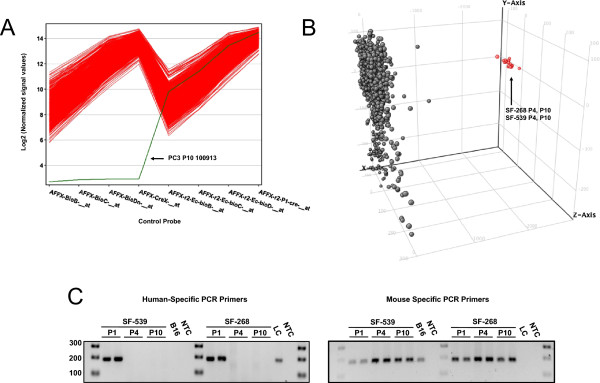
**Study design and quality control. A)** Control probe signal profiles were generated for 844 Affymetrix HG-U133 Plus 2.0 array .CEL files. The single outlier (PC-3 P10 100913) is highlighted. **B)** 3D principal component analysis (PCA) was performed on all .CEL files; a population of outliers representing P4 and P10 passages for SF-268 and SF-539 glioma lines is shown in red. Control probe profiles and 3D PCA were generated using Genespring GX11 (Agilent, Santa Clara, CA). **C)** Endpoint PCR of genomic DNA from SF-539 and SF-268 tumors at P1, P4 and P10 using mouse or human-specific *PTGER2* primers [see Methods]. Genomic DNA from B16F10 [B16, Mouse] and LnCAP [LC, human] cell lines were included as positive controls, NTC = no template control. Data is representative of all tumors processed from these xenografts.

Multiple layers of quality control criteria were applied to the dataset. First, the 844 CEL files (including P4 and P10 of SF-268 and SF-539) were visually inspected using DChip (http://www.hsph.harvard.edu/cli/complab/dchip/) to identify arrays with structural defects. Significant physical issues were identified in one MDA-MB-231 T P4 sample, which was removed. Next, .CEL files were uploaded and analyzed *en masse* using the QC tools contained within Genespring GX11 (Agilent, Santa Clara, CA). Results demonstrated one replicate from the PC-3 P10 had control probe errors and it was excluded (Figure 1A). The hybridization was repeated using the original PC-3 P10 RNA sample on a new chip that yielded satisfactory results and was included in further analyses. Lastly, principal component analysis (PCA) was used to identify outliers. For example, results shown in Figure 1B identify one population of arrays segregated from the primary component. Closer inspection identified these .CEL files as passages 4 and 10 of SF-268 and SF-539. This finding suggested a conserved problem with late passage for these lines. We then investigated whether displacement of tumor cells by an outgrowth of mouse cells was responsible for the observed effects. Results from endpoint PCR (Figure 1C) using mouse and human specific primers directed against *PTGER2*[14] showed the first *in vivo* passage of SF-268 and SF-539 tumors contained both mouse and human genomic DNA as expected, but all tumors harvested from 4th and 10th serial passages were devoid of human genomic DNA. The SF-268 and SF-539 models differed from others in the study in that they utilized Matrigel® to initially establish tumor growth in passage 1 and they were implanted into NOD.SCID/NCr mice since growth in athymic nu/nuNCr mice was unsuccessful. It is recognized that NOD.SCID/NCr mice have a propensity for spontaneous tumor formation [15] and also it is reasonable to speculate that Matrigel® (a growth-factor rich gel secreted by EHS (Engelbreth-Holm-Swarm) mouse sarcoma cells) may encourage spontaneous host cell outgrowth. This emphasizes the need for routine monitoring of mouse outgrowth especially when Matrigel®, and possibly a NOD.SCID background are employed. Although mouse outgrowth was not a problem for the remaining models, this phenomenon should be an important consideration for studies involving serial passage of tumor fragments over extended periods of time. In light of these results, P4 and P10 samples for SF-268 and SF-539 were not included in this data release.

### Hierarchical clustering QC

All experiments were subjected to hierarchical clustering to confirm similarity in microarray signatures for cell lines and subsequent xenograft passages. Fold-change data [100-fold cut-off] for each parameter was used to generate the hierarchical cluster shown in Additional file 1. This analysis confirmed that for the majority of experiments, the originating cell lines and subsequent *in vivo* passages clustered together. However, P10 data for CAKI-1, SN12C and RXF 393 renal lines were shown to cluster together, suggesting a high degree of similarity between these samples (Additional file 2A). The identity of these tumors was confirmed by repeating the Identifiler® STR analyses. Thus, the clustering of these P10 renal tumors of distinct cell line origins had a biological basis and was not the result of technical errors. Other exceptions involved the co-clustering of MDA-MB-435 and MDA-N (Additional file 2B) along with a similar trend for A549 and A549/Asc-1 (Additional file 2C). These observations were anticipated given that MDA-N is derived from MDA-MB-435 and A549/Asc-1 is a tumorigenic clone of A549. Additionally, two breast cancer cell lines (MCF7 and MDA-MB-231 T) did not cluster together nor did three ovarian lines (OVCAR-3, CP70, and OVCAR-5) (Additional file 2D and E). Closer scrutiny provides a plausible explanation given these lines differ significantly within their categories. Specifically, MCF7 is an estrogen-dependent (ER+) tumor whereas MDA-MB-231 T is triple negative (negative for estrogen receptor, progesterone receptor, Her2/neu). Similarly, CP70 and OVCAR-5 are ovarian carcinomas whereas OVCAR-3 is an adenocarcinoma. OVCAR-3 is also ER + while OVCAR-5 is ER negative and CP70 is a cisplatin-resistant subline of A2780. Following quality control, the final dataset comprised 47 complete experiments and 2 partial experiments (P0 and P1 for SF-268 and SF-539) for a total of 823 arrays.

### Monitoring changes in transcript expression

**Figure 2:**
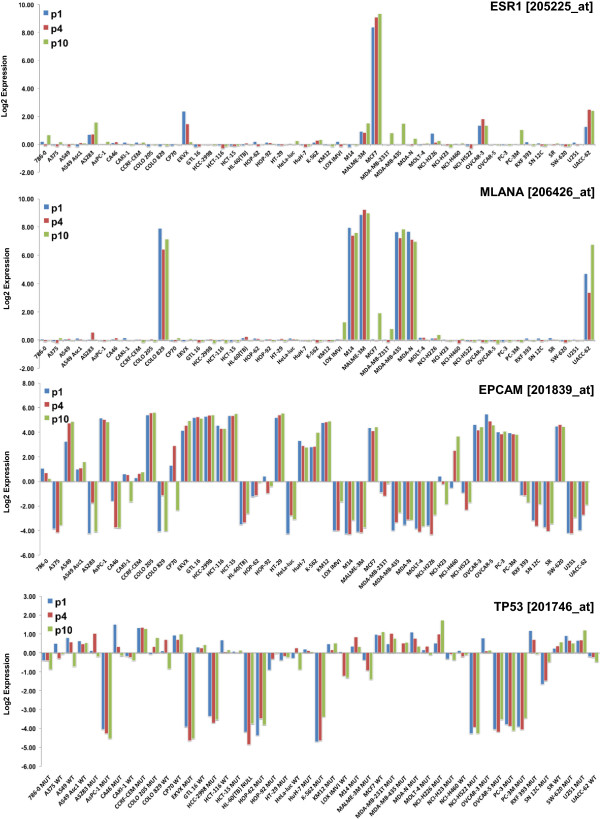
**Transcript expression relative to other models and with passage.** Log2 normalized gene expression values at passage 1, 4 and 10 plotted for four probe sets; 205225_at (ESR1, estrogen receptor alpha), 206426_at (MLANA, melan-A), 201839_s_at (EPCAM, epithelial cell adhesion molecule), and 201746_at (TP53, p53). The p53 status is shown for 201746_at as wild type (WT), mutant (MUT) or absent (NULL).

The central goal of this study was to ascertain expression levels for any given transcript across all *in vivo* models and with successive passage. To this end, normalized gene expression data were generated for all 192 conditions [see Additional file 3, txt file should be copied into EXCEL or other spreadsheet application to view]. Throughout the study, when multiple probe sets were present for the same transcript, the Jetset methodology [16] was used to select the most robust candidate. Data for four example probe sets; 205225_at (ESR1, estrogen receptor alpha), 206426_at (MLANA, melan-A), 201839_s_at (EPCAM, epithelial cell adhesion molecule), and 201746_at (TP53, p53) at P1, P4 and P10 are plotted in Figure 2. Results showed that MCF7 cells expressed the highest level of ESR1 across the panel, consistent with their known estrogen receptor positive status and their absolute dependence on exogenous estradiol for growth in mice. Furthermore, ESR1 expression was shown to increase modestly with passage, suggesting serially passaged tumors remain a valid target for estrogen receptor antagonists. MLANA (Melan-A) is a melanoma-restricted antigen and results show expression to be limited primarily to the melanoma cell lines. MLANA expression was relatively consistent with passage, apart from UACC-62 where levels modestly increase at P10. Similarly, EPCAM is highly expressed on many epithelial cells and results show considerable variation in mRNA expression levels even between epithelial lines. While most tumors had stable expression of EPCAM from P1 to P10, there were a few models with notable changes including A549, CaKi-1 and NCI-H460. The final example, TP53/p53 serves to show the value of integrating relative expression data with pre-existing genetic information. This tumor suppressor and master regulator of the cell cycle is frequently mutated or deleted in tumors [17, 18]. This analysis confirmed the relative lack of p53 expression in p53-null HL60 cells. However, there was considerable variability in levels of expression for models with mutant p53. This information could be useful for studies of agents targeting cells with mutant/deleted p53 e.g., the Wee1 inhibitor MK-1775 or mutant p53 oncolytic adenovirus [19, 20]. Overall, this analysis serves to illustrate the potential of these data within therapeutic studies and provide evidence that mRNA expression can change markedly with *in vivo* passage.

**Figure 3:**
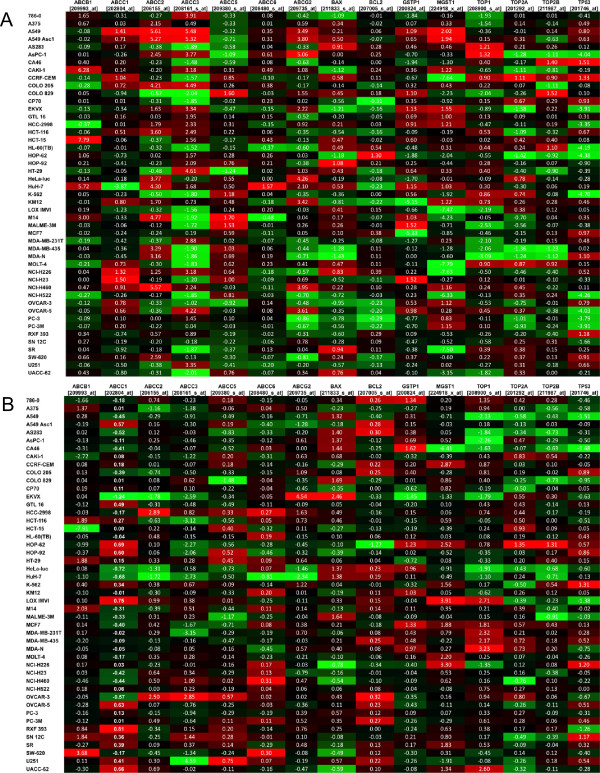
**Change in expression from P1 to P10 for a subset of transcripts involved in drug resistance. A)** Log2 normalized expression values for each transcript in all models at P1. **B)** Change in log2 normalized expression for each transcript in all models from P1 to P10. For each probe set, entries are formatted where red is the highest value, green is the lowest and the median is black.

Another approach to data mining involves plotting the extent of change in expression with passage. To exemplify, analyses were performed on two groups of therapeutically relevant transcripts, those involved in drug resistance and those coding for select receptor tyrosine kinases (RTKs). For resistance genes, expression relative to other models at P1 was plotted (Figure 3A) along with the change in expression from P1 to P10 (Figure 3B). Several noteworthy trends were evident. For HCT-15, a highly MDR drug-resistant cell line in culture, expression of the multidrug transporter ABCB1 [MDR1] was the highest relative to other lines at P1. However by P10, the expression had declined to that comparable to other tumors. This suggests HCT-15 tumors may acquire sensitivity to agents such as doxorubicin, paclitaxel and other MDR sensitive agents with serial *in vivo* passage [21]. This finding is confirmed by *in vivo* efficacy data for paclitaxel against subcutaneously implanted HCT-15 xenografts (Additional file 4). As suggested by the array data, differences in the response of P1 and P8 tumors to paclitaxel treatment were observed. The P1 tumors showed progressive growth in spite of paclitaxel therapy (Additional file 4A). In contrast, the P8 tumors responded to treatment with total growth inhibition (Additional file 4B). Comparison of tumor weights between the P8 vehicle and P8 paclitaxel-treated mice showed statistically significant differences at days 22, 26 and 29 with p values of 0.0004, 0.001, and 0.0001, respectively. In contrast, there was no difference between the P1 vehicle-treated and paclitaxel-treated groups at any time point. These data support the conclusion that HCT-15 human colon tumor xenografts acquire paclitaxel sensitivity during serial *in vivo* passage.

Expression of microsomal glutathione S-transferase (MGST) has been shown to confer resistance to cisplatin [22]. In this analysis, expression of MGST1 [224918_x_at] increased with passage in several cell lines such as LOX IMVI and NCI-H226. Given the general trend toward increased expression from P1 to P10, it could be surmised that increased resistance would be observed in serially passaged tumors. This observation is consistent with our data, where evaluation of cisplatin in subcutaneous LOX IMVI xenografts showed sensitivity to cisplatin declines with serial *in vivo* passage. When cisplatin was administered at the maximum tolerated dose to LOX-IMV1 tumor-bearing mice at P1, P4 and P10, the optimal percent test/control values were 19%, 41%, and 64%, respectively. The %T/C is inversely related to the tumor sensitivity so the greater the tumor response the lower the %T/C. A %T/C of 40% or lower is indicative of antitumor activity [23]. Thus, as predicted by the changes in MGST1 relative expression in LOX IMV1 tumors with serial *in vivo* passage, P1 tumors were highly sensitive while the P10 tumors were not. The ranking of relative MGST1 expression levels at P1 shows A549/Asc-1 and A549 have the greatest and MOLT-4 the lowest MGST1 expression. This suggests cisplatin would be inactive against A549 tumors and active against MOLT-4 tumors. This is borne out by *in vivo* sensitivity testing where MOLT-4 has a statistically significant reduction in tumor growth in mice receiving 3.24 mg cisplatin/kg compared to vehicle controls (Additional file 5A) while A549 tumors do not respond even with an increased cisplatin dose (6.7 mg/kg) and a smaller initial starting tumor size (Additional file 5B).

Interestingly, the reverse trend in mRNA expression was observed in CA46 cells, where MGST1 was downregulated at P10. Here, although no xenograft data exists to confirm this, sensitivity to cisplatin could be predicted to increase with *in vivo* passage. Lastly increased expression of ABCG2, a transporter that enhances resistance to mitoxantrone, daunorubicin and doxorubicin [24] was noted in EKVX cells from P1 to P10.

**Figure 4:**
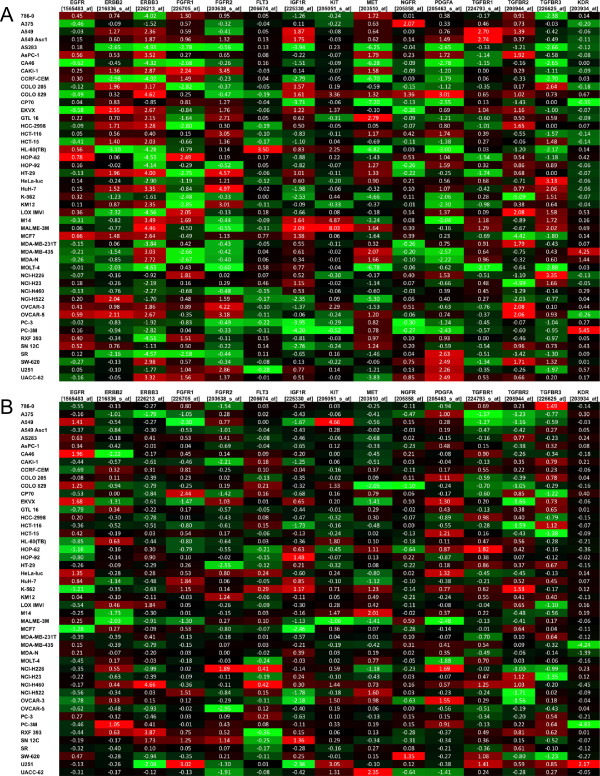
**Change in expression from P1 to P10 for a subset of transcripts coding for receptor tyrosine kinases (RTKs). A)** Log2 normalized expression values for each transcript in all models at P1. **B)** Change in log2 normalized expression for each transcript in all models from P1 to P10. For each probe set, entries are formatted where red is the highest value, green is the lowest and the median is black.

Results in Figure 4 show a similar analysis focused on a subset of cancer-related receptor tyrosine kinases. Again, several trends were evident. For example, PC-3 M cells expressed the highest relative levels of KDR (VEGFR2; 203934_at) at P1. However, levels declined by P10, an observation that could have consequences for evaluation of therapeutics targeting this pathway (e.g., sorafenib). The melanoma cell lines M14 and MALME-3 M expressed significant levels of KIT (stem cell factor receptor) [205051_s_at] relative to other models at P1. With serial passage, expression of KIT declined slightly in MALME-3 M by P10 whereas the levels were increased in M14 at P10. Conversely, A549 cells expressed low levels of KIT at P1, but expression increased markedly at P10. These observations may have consequences for experiments where a c-Kit inhibitor (e.g., imatinib) is being evaluated.

### A general metric of model stability

**Figure 5:**
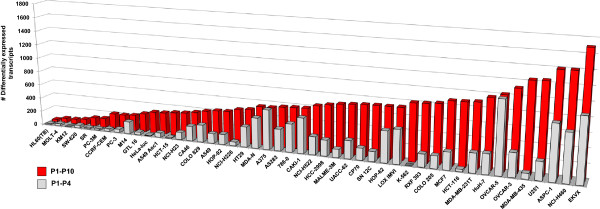
**Ranking model stability.** For each model, Genespring GX11 was used to generate a list of differentially expressed transcripts for P1 to P4 and P1 to P10 [3-fold cut-off, p < 0.05]. The number of differentially regulated transcripts at P1 to P4 and P1 to P10 was then plotted for each model and results sorted in terms of P1 to P10 [lowest to highest] to generate a measure of model stability.

The prior analysis suggested that significant changes in transcript expression occurred in several models from P1 to P10. Therefore, we sought a general metric of stability for each xenograft model. To achieve this, the number of transcripts showing >3-fold increase or >3-fold decrease (p < 0.05) from P1 to P4 and P1 to P10 was determined for each of 47 models. We reasoned the higher the stability of the model, the smaller the number of transcripts with changes in expression that would be observed between passages. Figure 5 illustrates the results of this analysis, where models such as HL-60 (TB) and MOLT-4 (32 and 66 differentially-expressed transcripts between P1 and P10, respectively) may be defined as highly stable, whereas NCI-H460 and EKVX tumors are markedly altered at P10 (1401 and 1696 differentially expressed transcripts between P1 and P10, respectively) with approximately 50% of these changes occurring between P1 and P4. From this analysis it is evident that protracted *in vivo* growth profoundly alters the transcriptome for most models. Generation of a simple metric of stability allows models to be ranked according to the degree of change. Whether these changes represent gradual adaptations to growth in a mouse microenvironment or selection-pressure promoting outgrowth of sub-clones remains to be determined. However, it is interesting to note that a survey of genes (CDH1, FN1, KRT19) related to the epithelial-mesenchymal transition (EMT) identified widespread changes in several models with passage (results not shown). Given the recognized effects of EMT on drug sensitivity, this phenomenon may be worthy of further scrutiny [25].

### The cell line to xenograft transition

This dataset can be interrogated to monitor gene expression changes occurring during the immediate transition from *in vitro* to *in vivo* growth. However, this analysis is complicated by potential interference from cross-hybridizing mouse mRNA from the xenograft samples. Although cell sorting can remove mouse cell contamination [26], an alternative strategy involves identifying probe sets on the Affymetrix HG-U133 Plus 2.0 array with the potential to cross-hybridize with mouse mRNA and excluding these from analysis or flagging them for additional evaluation. To this end, 5 murine mRNA samples (mouse universal RNA, B16F10 murine melanomas and skin from the C57BL/6 mice bearing the tumor, colon 26 murine tumors and skin from the Balb/c mice bearing the tumor), all performed in triplicate, were applied to the human Affymetrix HG-U133 Plus 2.0 arrays to generate a list of cross-hybridizing probe sets [see Methods]. Mouse RNAs of varied origin were used to provide diversity in the detection of cross-hybridizing events. Results demonstrated a total of 7963 probe sets have the potential to bind mouse mRNA. This includes all possible binding events ranging from just one of the mouse samples cross hybridizing to a probe set to the cross hybridization of all five of the different murine samples. Although relatively large, this list likely includes probe sets that bind non-specifically to any cDNA population along with those mRNAs showing high homology between mouse and human. Increasing the stringency to 4 and 5 out of 5 murine samples that must bind to a particular probe set before that probe is excluded from the analysis reduces the number of probe sets with the potential to cross-hybridize to murine RNA by 3-fold (to 2614). To provide flexibility to the investigator the list of transcripts with the total number of mouse RNA samples found to cross-hybridize to each probe set is shown in Additional file 6.

As an example of the *in vitro* to *in vivo* transition, Table 2 shows the results from pairwise analysis of prostate lines (PC-3 and PC-3/M at P0) with their respective P1 xenografts. The 50 highest up-regulated transcripts before removal of the 14 cross hybridizing probe sets are shown in Table 2, with transcripts showing potential mouse artifacts identified. Hemoglobin epsilon 1 (HBE1) [217683_at] is a probable cross-hybridization event that is important because PC-3 cells are not expected to express this mRNA. Other genes of note that were removed as a result of mouse cross-hybridization include SERPINB6, CXCL1, COL3A1 and ADM. All with the exception of SERPINB6 are genes of the stromal compartment [27–29]. Removal of the mouse cross-hybridizing probes provides greater confidence that observations such as increased expression of IL-8 (202859_x_at), CXCL6 (206336_at), and ANGPTL4 (223333_s_at) represent genuine adaptations of PC-3/PC3-M cells to *in vivo* growth. These upregulated genes point toward activation of pathways involved in invasion and metastasis [30–32]. Table 3 shows DAVID gene ontology (GO) analysis for the top 50 up-regulated genes following removal of the mouse cross-hybridizing probes. Consistent with the literature [33–37] genes involved in the extracellular matrix (ECM), cell adhesion, chemotaxis, cytokine, immune response, tumor-host interaction and growth factor induced signal transduction were up-regulated in xenograft tumors relative to those cells grown *in vitro*. A spreadsheet of fold-change data for the P0 to P1 transition (with cross-hybridizing transcripts noted) is shown in Additional file 7. Similar analyses for the P0 to P1 transition for the top 50 up-regulated genes and respective DAVID GO analysis for 9 of the 13 model classes (see Table 1) can be found in Additional file 8.

**Table 2:** **Top 50 up-regulated probe sets in the prostate tumor type**

Prostate mouse & human probe set ID	Prostate gene symbol	Prostate P0->P1 average fold change	# Mouse cross-Hyb tissues	Prostate human probe set ID	Prostate gene symbol	Prostate P0->P1 average fold change
231628_s_at	SERPINB6	8.169	5	202859_x_at	IL8	5.290
216405_at		6.239	5	206336_at	CXCL6	5.190
217683_at	HBE1	6.171	5	209183_s_at	C10orf10	4.672
202859_x_at	IL8	5.290		223333_s_at	ANGPTL4	4.479
206336_at	CXCL6	5.190		211506_s_at	IL8	4.451
209183_s_at	C10orf10	4.672		201438_at	COL6A3	4.328
204470_at	CXCL1	4.551	1	212977_at	CXCR7	4.231
223333_s_at	ANGPTL4	4.479		1570537_a_at		4.170
211506_s_at	IL8	4.451		211756_at	PTHLH	4.153
201438_at	COL6A3	4.328		203828_s_at	IL32	3.987
217572_at		4.301	5	241436_at	SCNN1G	3.967
212977_at	CXCR7	4.231		214157_at	GNAS	3.756
1570537_a_at		4.170		221009_s_at	ANGPTL4	3.744
211756_at	PTHLH	4.153		213711_at	KRT81	3.485
203828_s_at	IL32	3.987		206300_s_at	PTHLH	3.460
241436_at	SCNN1G	3.967		1569978_x_at		3.456
215076_s_at	COL3A1	3.775	5	211071_s_at	MLLT11	3.355
202912_at	ADM	3.763	1	201578_at	PODXL	3.249
214157_at	GNAS	3.756		222449_at	PMEPA1	3.230
221009_s_at	ANGPTL4	3.744		205199_at	CA9	3.189
224344_at	COX6A1	3.700	5	201890_at	RRM2	3.106
213711_at	KRT81	3.485		211030_s_at	SLC6A6	3.100
201852_x_at	COL3A1	3.483	5	222608_s_at	ANLN	3.062
206300_s_at	PTHLH	3.460		210095_s_at	IGFBP3	3.028
1569978_x_at		3.456		207291_at	PRRG4	3.006
211071_s_at	MLLT11	3.355		209774_x_at	CXCL2	2.979
201578_at	PODXL	3.249		211161_s_at	COL3A1	2.974
222449_at	PMEPA1	3.230		206157_at	PTX3	2.970
205199_at	CA9	3.189		203373_at	SOCS2	2.969
201890_at	RRM2	3.106		232381_s_at	DNAH5	2.929
211030_s_at	SLC6A6	3.100		212143_s_at	IGFBP3	2.928
1570107_at		3.091	5	238513_at	PRRG4	2.922
222608_s_at	ANLN	3.062		201291_s_at	TOP2A	2.911
210095_s_at	IGFBP3	3.028		202404_s_at	COL1A2	2.908
207291_at	PRRG4	3.006		205479_s_at	PLAU	2.892
209270_at	LAMB3	3.002	2	205680_at	MMP10	2.803
209774_x_at	CXCL2	2.979		217875_s_at	PMEPA1	2.797
211161_s_at	COL3A1	2.974		230280_at	TRIM9	2.763
206157_at	PTX3	2.970		227556_at	NME7	2.723
203373_at	SOCS2	2.969		242517_at	KISS1R	2.699
232381_s_at	DNAH5	2.929		218691_s_at	PDLIM4	2.688
212143_s_at	IGFBP3	2.928		219148_at	PBK	2.684
238513_at	PRRG4	2.922		203691_at	PI3	2.684
201291_s_at	TOP2A	2.911		202998_s_at	LOXL2	2.681
202404_s_at	COL1A2	2.908		210538_s_at	BIRC3	2.669
223484_at	C15orf48	2.896	1	229435_at	GLIS3	2.667
205479_s_at	PLAU	2.892		41469_at	PI3	2.649
227140_at	INHBA	2.830	1	218355_at	KIF4A	2.624
211668_s_at	PLAU	2.829	1	214438_at	HLX	2.623
205680_at	MMP10	2.803		209156_s_at	COL6A2	2.597

Left table includes all probe sets, the mouse RNA binding probe sets are identified by the number of cross hybridizing mouse tissue probes that occurred (1-5 see column 4). Right side of table shows the top 50 human up-regulated probe sets following removal of the mouse component.

**Table 3:** **DAVID Gene Ontology (GO) functional annotation on the prostate tumor models during their transition from in vitro to in vivo growth**

Accession	Gene Ontology Term generated from DAVID analysis of human-RNA-only-binding probe sets	Gene count	PValue	Fold enrichment	Benjamini	False discovery rate (%)
GO:0044421	extracellular region part	14	4.68E-07	5.33	4.12E-05	5.07E-04
GO:0005615	extracellular space	12	8.91E-07	6.40	3.92E-05	9.65E-04
GO:0005576	extracellular region	18	3.26E-06	3.27	9.55E-05	3.53E-03
GO:0005578	proteinaceous extracellular matrix	7	1.75E-04	7.99	3.84E-03	0.19
GO:0031012	extracellular matrix	7	2.63E-04	7.41	4.61E-03	0.28
GO:0009611	response to wounding	7	0.0019	5.10	0.6674	2.70
GO:0019932	second-messenger-mediated signaling	5	0.0027	8.22	0.5559	3.96
GO:0001568	blood vessel development	5	0.0032	7.89	0.4668	4.58
GO:0001944	vasculature development	5	0.0035	7.70	0.4022	4.99
GO:0005581	collagen	3	0.0039	31.30	0.0553	4.11
GO:0048870	cell motility	5	0.0071	6.30	0.4510	9.91
GO:0051674	localization of cell	5	0.0071	6.30	0.4510	9.91
GO:0008009	chemokine activity	3	0.0072	22.88	0.6673	8.26
GO:0006935	chemotaxis	4	0.0074	9.66	0.4245	10.41
GO:0042330	taxis	4	0.0074	9.66	0.4245	10.41
GO:0001501	skeletal system development	5	0.0081	6.06	0.4132	11.24
GO:0042379	chemokine receptor binding	3	0.0081	21.48	0.4633	9.29
GO:0005125	cytokine activity	4	0.0166	7.20	0.5732	18.13
GO:0005201	extracellular matrix structural constituent	3	0.0236	12.24	0.5992	24.90

## Conclusions

The evaluation of cancer therapeutics using xenograft models is a resource-intensive and time-consuming endeavor. Currently, molecular profiling with large-scale genetic, proteomic and metabolic screening technologies is emerging as a powerful tool to improve and guide model selection. In this study, cDNA microarray data was generated for 49 xenograft models routinely used within the Developmental Therapeutics Program at the NCI. The majority of these tumor cell lines are available to the research community through the DCTD Tumor Repository (NCI at Frederick) thus allowing this data to provide guidance for other research studies. Aside from the obvious utility of ranking models in terms of transcript expression for specific genes of interest, analysis demonstrated that prolonged *in vivo* passage markedly alters the transcriptome of many models. This observation emphasizes the importance of maintaining passage consistency to minimize experimental artifacts. Similarly, these findings provide one explanation for the difficulties reported in reproducing experimental data since the methods for generating test tumors can impact the study profoundly [38]. These data are deposited at http://dtp.nci.nih.gov/microxeno/download.html and in the Gene Expression Omnibus (http://www.ncbi.nlm.nih.gov/geo/) under accessions GSE48433 and GSE49353. All subsequent data releases will be deposited in the same locations.

## Methods

### Cell lines and mice

All cell lines were obtained from the Division of Cancer Treatment and Diagnosis Tumor Repository (DTP, National Cancer Institute at Frederick, Frederick, MD) or American Type Culture Collection (ATCC) (Manassas, Va). In addition, the identities of all cell lines used in this study were confirmed using Identifiler® STR genotyping (Applied Biosystems). All mice used in the study were obtained from the Animal Production Program (National Cancer Institute at Frederick, Frederick, MD). All studies were conducted in an Association for Assessment and Accreditation of Laboratory Animal Care International (AAALACi) accredited facility under a protocol approved by the NCI at Frederick Animal Care and Use Committee. This facility operates under an Office of Laboratory Animal Welfare (OLAW) assurance in compliance with the U.S. Public Health Service Policy on Humane Care and Use of Animals (1996) and the Guide for the Care and Use of Laboratory Animals Eighth Edition. PCR primers were purchased from ABI (Applied Biosystems). Karyotype analysis and STR genotyping results for most seed lines are available at http://dtp.cancer.gov/branches/btb/characterizationNCI60.html.

### Tumor growth, propagation and sampling

Tumor cells for inoculation were derived from the 4th *in vitro* passage from cryopreserved cell stocks. Cells (1 × 10^7^ cells/0.1 ml/inoculation) were inoculated subcutaneously into mice (n = 10 or more mice depending on the tumor cell line). For comparative purposes, 2–3 samples of *in vitro* cultivated cells at passage 4 (same as used for *in vivo* implantation) were also prepared for microarray analysis (P0). When tumors at passage 1 (P1), passage 4 (P4) and passage 10 (P10) reached approximately 500 mg (10×10 mm), 5 tumors were harvested, cut into small pieces and flash frozen in liquid nitrogen using pre-chilled cryovials. Samples were transferred to a -70°C freezer for holding prior to processing. Serial tumor passage was achieved by harvesting donor tumor material (n = 2 to 5 donors) at each passage, mincing the tumors (2x2 mm fragments), pooling the samples and inoculating the recipients subcutaneously using tumor implant trocars. Athymic nude mice (athymic NCr-nu/nu) served as the primary host for most xenograft studies. In several instances [particularly with human leukemias and lymphomas] it was difficult to establish and passage tumors in athymic nude mice. In these instances, SCID/NCr or NOD.SCID/NCr mice were assessed for suitability. In general, female mice were used as they are less aggressive, easily group-housed and are routinely used in xenograft experiments. Male mice were used for prostate tumors (PC-3, PC-3/M). Two tumors (SF-268 and SF-539) required the use of Matrigel™ (Collaborative Research, Bectin Dickinson, Bedford, Ma) during the initial tumor cell inoculation to achieve progressive tumor growth. Finally, in those instances where hormone effects on tumor growth are important (e.g., estradiol-dependent breast cancers) mice were supplemented with estradiol (up to 3 mg/kg) administered subcutaneously once weekly to maintain progressive tumor growth. Exceptions to the standard protocol are noted in Table 1.

### Comparison of HCT-15 sensitivity to paclitaxel at early and late *in vivo* passage

*In vivo* antitumor activity assays were performed with six-week-old female athymic nude mice (Animal Production Program, Frederick, MD). Forty mice were each implanted subcutaneously with 1x10^7^ HCT-15 human colon cancer cells (DCTD Repository, Frederick, MD). Thirty of these passage 1 (P1) tumor-bearing mice were randomized into 2 groups (n = 20/vehicle control group and 10/treated group) and the remaining tumors were used to serially passage the tumors. For this, tumors were harvested from donor mice, cut into fragments of 2–3 mm and the resulting fragments were implanted subcutaneously into recipient mice using a tumor implant trocar. This process was repeated when the tumors reached 500–750 mg until the tumors reached their eighth *in vivo* passage (P8). At P8, tumor-bearing animals were randomized into 2 groups (n = 18/vehicle control group and 9/treated group). At P1 and P8 the vehicle control groups received 12.5% cremaphor/12.5% ethanol/75% saline while the treated group received 10 mg paclitaxel/kg. Both treatments were administered intravenously (IV) once daily for 5 days (QDx5) using a standard dosing volume of 10 ml/kg body weight. Tumor growth was monitored by caliper measurements, and tumor weights were calculated as: [tumor length in mm × (tumor width^2^ in mm)]/2 = weight in mg (1). Tumor measurements were collected every 3 to 4 days throughout the course of the study. Student’s t-test was used to assess the significance in response of the paclitaxel treated animals to the corresponding controls with significance set at a p value of 0.05 or less. The studies were terminated 12 – 15 days after the last drug dose.

### *In vivo* sensitivity testing of MOLT-4 and A549 xenografts to cisplatin treatment

*In vivo* antitumor activity assays were performed with six-week-old female athymic nude mice (Animal Production Program, Frederick, MD). MOLT-4 and A549 tumors were established in cohorts of mice for evaluation of cisplatin sensitivity. When MOLT-4 tumors reached a median of 150 mg the mice were randomized into vehicle control (n = 20) and cisplatin treated groups (n = 10). The vehicle control group received 0.9% saline while the cisplatin was administered at 3.24 mg/kg. When A549 tumors reached a median of 110 mg the mice were randomized into vehicle control (n = 15) and cisplatin-treated groups (n = 6). The vehicle control group received 0.9% saline while the cisplatin was administered at 6.7 mg/kg. All treatments were administered intraperitoneally (IP) at one dose per day every fourth day for a total of 3 doses (Q4Dx3) using a standard dosing volume of 10 ml/kg body weight. Tumor growth was monitored as noted above. The studies were terminated when control tumor weights exceeded 2500 mg or 3 weeks post-last drug dose, whichever occurred first.

### Tumor homogenization and RNA Isolation

Frozen xenograft tumor specimens were removed from the freezer and kept in liquid nitrogen. Tumors were cut into smaller pieces using a pill cutter pre-chilled by submersion into liquid nitrogen. A tumor fragment was placed into the pill cutter and quickly cut into smaller pieces (approximately 10–25 milligrams each). The tumor samples were homogenized in RLT-ME buffer (Qiagen, Germantown, MD) using a single 5 mm steel bead per sample in a Tissuelyser (Qiagen, Germantown, MD) for two minutes at 28 Hz. The volume of RLT-ME buffer for each sample was calculated using a standard formula of 20 μL RLT-ME per mg of tissue. Samples were incubated at RT for 10 min after homogenization following which the tumor material was further homogenized using a QiaShredder column. The RNA was extracted using RNeasy® Mini Kits (Qiagen, Germantown, MD) following the manufacturer’s protocol. The tumor cells (5-7 × 10^6^) were harvested from sub-confluent flasks at passage 4 by scraping. Following centrifugation, the cell pellets were flash-frozen in liquid nitrogen and stored at -80°C. RNA from the cell pellets was isolated using the RNeasy® Mini Kit following the manufacturer’s protocol.

### RNA quantitation and quality control

Following extraction, RNA quantitation was performed using a Nanodrop spectrophotometer (Thermo Scientific, Wilmington, DE). Microelectrophoresis was performed on all samples using an Agilent Bioanalyzer (Santa Clara, CA) to assess sample quality. The Agilent software algorithm evaluates the entire electrophoretic trace to estimate the total RNA integrity in a sample. The algorithm calculates an RNA Integrity Number (RIN), which classifies the quality of the RNA on a numeric system from 1 to 10, with 1 being the most degraded and 10 being the most intact. The RIN number allows comparison of the quality of the RNA between samples and ensures better reproducibility. In this study, 73% of RNA samples scored a RIN of >9, while 25% had a RIN of 7–9 and 2% had a RIN 5.7-7.

### Microarray analysis

The RNA samples were submitted to Expression Analysis, Inc. (Durham, NC) for microarray analysis using the HG-U133 Plus 2.0 array (Affymetrix, Santa Clara, CA). Samples were prepared and hybridized according to the “Affymetrix Technical Manual” (http://www.expressionanalysis.com). Following staining of the microarray, the fluorescent images were detected in a GeneChip® Scanner 3000 and expression data were extracted using the GeneChip Command Console Software (AGCC) v 2.0 (Affymetrix). All GeneChips were scaled to a median intensity setting of 500. Raw array (.CEL files) data are available for direct download from the Developmental Therapeutics Program (http://dtp.nci.nih.gov/microxeno/download.html) and from the Gene Expression Omnibus (http://www.ncbi.nlm.nih.gov/geo/), accessions GSE49353 and GSE48433.

Microarray data were analyzed using the default ‘guided workflow’ option within Genespring 12.6.1 (Agilent, Santa Clara, CA). In brief, raw data (.CEL) files were imported and replicates assigned to relevant conditions (e.g. 786–0 P0). All data files within the experiment were then normalized using RMA [39], to generate a spreadsheet containing log2 normalized gene expression values. Next, probe sets were filtered with reference to flags [attributes that denote poor quality entities] along with a percentile cut-off (20%) - which makes the assumption that 20% of the probe sets on any given genome-wide array have intensity values that represent noise (since they are not expressed). Next, significance was determined by performing one-way ANOVA p < 0.05. For the remaining observations a fold change cut-off could then be applied [default +/- 2 fold].

### Mouse/human endpoint PCR

As described in Alcoser *et al*. [14] human- or mouse-specific forward primers from the prostaglandin E receptor 2 (*PTGER2*) gene were used to amplify 189 bp fragments, thereby identifying the presence of mouse DNA and/or human DNA in each tissue/cell sample. Human-specific primers: Forward, 5′- gctgcttctcattgtctcgg-3′; Reverse, 5′- gccaggagaatgaggtggtc-3′. Mouse-specific primers: Forward, 5′- cctgctgcttatcgtggctg-3′; Reverse, 5′- gccaggagaatgaggtggtc-3′. PCR was performed using neutralized, unpurified tissue/cell lysate on an ABI-2720 Thermocycler (Applied Biosystems). PCR conditions: 95°C-5 min, 30 cycles of (95°C-45 sec, 60°C-30 sec, 72°C-90 sec), 72°C-10 min. DNA bands were resolved on a 2% agarose gel containing ethidium bromide (0.5 mg/ml).

### Identification of probe sets on Affymetrix U133 Plus 2 array with the potential to cross-hybridize with mouse transcripts

Array data were generated for 5 different mouse RNA populations hybridized to human Affymetrix U133 Plus 2 chips (3 replicates for each biological sample). The samples were A) mouse Universal RNA (Strategene, La Jolla, CA), B) B16F10 murine melanoma tumors, C) skin from C57BL/6 mice bearing B16F10 tumors, D) colon 26 murine tumors, and E) skin from Balb/C mice bearing Colon 26 tumors. Mouse RNAs of varied origin were used to provide a diversity of mouse transcripts for subsequent qualitative detection of array binding. The 15 .txt files generated (5 biological samples, 3 replicates each) were interrogated and for each sample type, all probe sets with a PPP, PPM or PPA [P = present, M = marginal, A = absent] detection call were sorted into a separate file (total 5 files). Each letter of the PPP or PPM, or PPA nomenclature represents 1 array from the triplicate samples. The five lists of probes were combined into a single file and the Microsoft Excel utility DigDb [http://www.Digdb.com] was used to remove redundancy and to determine how many biological samples contained detectable transcript (shown as PPX ID in # Mouse Tissues – out of five). The resultant list of probe sets and number of mouse RNA samples in which they are called present (PPX) can be found in Additional file 7.

### Availability of supporting data

The data sets supporting the results of this article are available for direct download from the Developmental Therapeutics Program (http://dtp.nci.nih.gov/microxeno/download.html) and from the Gene Expression Omnibus (http://www.ncbi.nlm.nih.gov/geo/), accessions GSE49353 and GSE48433 as well as in the Additional Files.

## Electronic supplementary material

Additional file 1: **Hierarchical clustering of cell line and xenograft samples.** 823 arrays [47 complete models] were uploaded into Genespring (Agilent, Santa Clara, CA) using the Affymetrix expression guided workflow and data normalized using RMA. Entities were then assigned and log2 gene expression data generated for those transcripts that met the following criteria: percentile cut-off = 20, ANOVA p < 0.05, probe sets must be differentially expressed +/- 100 fold in 1 group. The probe sets meeting these criteria were then subjected to Hierarchical clustering according to both conditions [cell lines/passages] and entities [probe sets] with a Pearson absolute distance metric and centroid linkage. (ZIP 5 MB)

Additional file 2: **Issues identified during Hierarchical clustering.** (PPTX 113 KB)

Additional file 3: **RMA normalized log2 gene expression data for all 49 models - unfiltered, includes all probe sets on the U133 Plus 2.0 Array.** (ZIP 17 MB)

Additional file 4: **Antitumor efficacy of paclitaxel against HCT-15 xenografts at passages 1 (P1) and 8 (P8).** Vehicle treated mice received 12.5% cremaphor/12.5% ethanol/75% saline QDx5 IV while paclitaxel was administered at 10 mg/kg QDx5 IV. Statistically significant differences between the treated and control mice were determined with Student’s t-test, those points with significant paclitaxel responses are designated by showing the p value adjacent to the data point. A) P1 tumors B) P8 tumors. (PPTX 112 KB)

Additional file 5: **Antitumor efficacy of cisplatin against MOLT-4 and A549 xenografts.** Vehicle treated mice received 0.9% saline once every 4 days for 3 treatments (Q4Dx3) by the intraperitoneal (IP) route. Statistically significant differences between the treated and control mice were determined with Student’s t-test, those points with significant cisplatin responses are designated by with the p value adjacent to the data point A) MOLT-4 xenografts, the cisplatin dose was 3.24 mg/kg. B) A549 xenografts, the cisplatin dose was 6.7 mg/kg. (PPTX 108 KB)

Additional file 6: **List of transcripts identified as cross-hybridizing with mouse cDNA.** (XLS 968 KB)

Additional file 7: **Change in expression (log2) across the entire U133 plus 2 array for all models from P0 to P1 with cross-hybridizing probe sets shown.** (ZIP 7 MB)

Additional file 8: **A) Top 50 up-regulated probe sets in 9 of 13 tumor types.** Left table includes all probe sets, where mouse cross-hybridizing probe sets are shaded grey. Right table shows the top 50 up-regulated probe sets following removal of the cross hybridizing component. B) DAVID gene ontology (GO) functional annotation for each tumor model during transition from *in vitro* to *in vivo* growth. (XLSX 80 KB)
