# Supplementary figures and images for: Gene expression profiling of 49 human tumor xenografts from in vitro culture through multiple in vivo passages - strategies for data mining in support of therapeutic studies
Source: BMC Genomics. 2014 May 22;15(1):393. doi: 10.1186/1471-2164-15-393 (PMC4041995; doi:10.1186/1471-2164-15-393)

## Slide 1
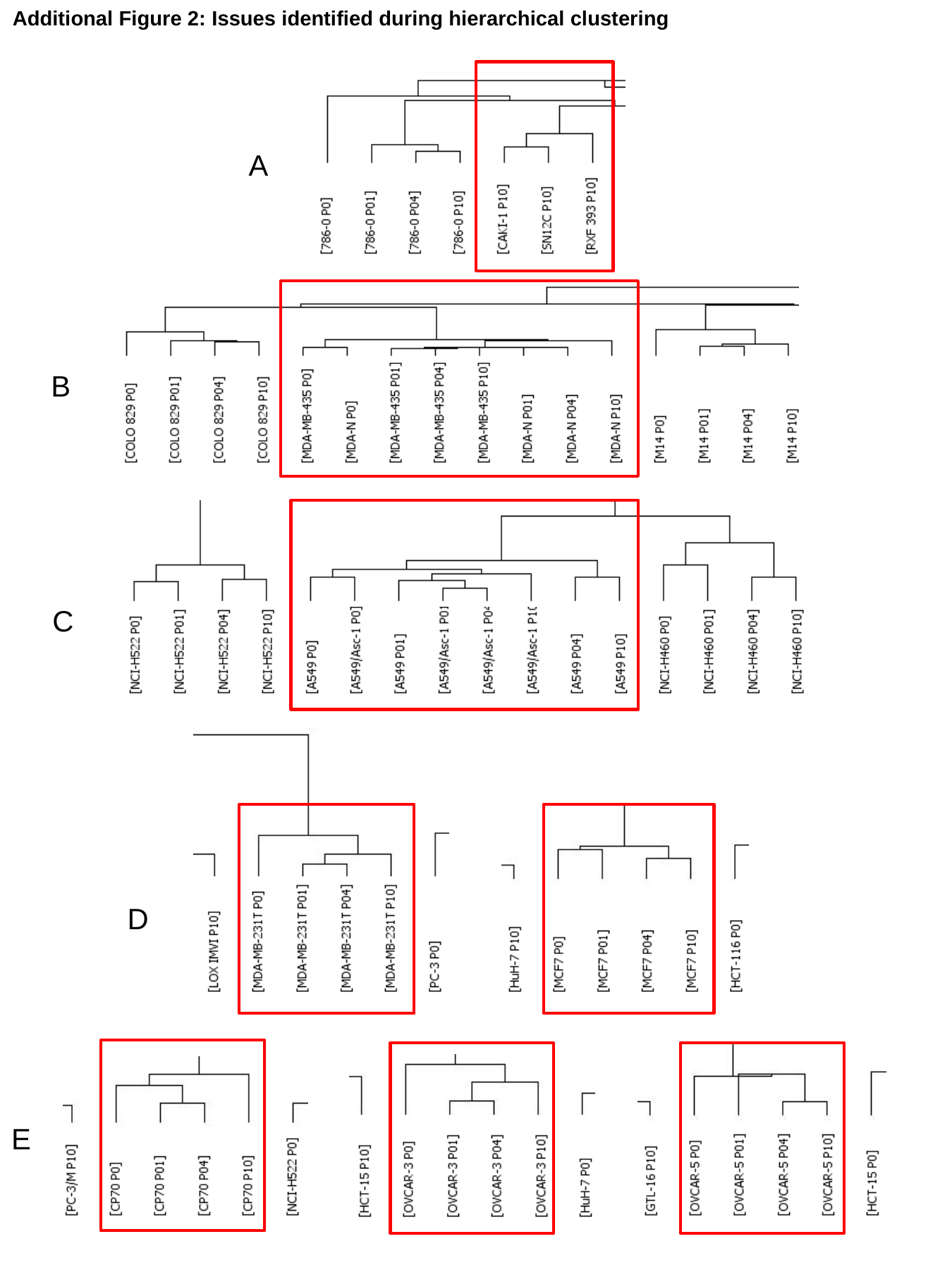

Additional Figure 2: Issues identified during hierarchical clustering
A
B
C
D
E

Supplement: Supplementary file 2 — Additional file 2: Issues identified during Hierarchical clustering. (PPTX 113 KB) [file 12864_2013_6082_MOESM2_ESM.pptx]

## Slide 1
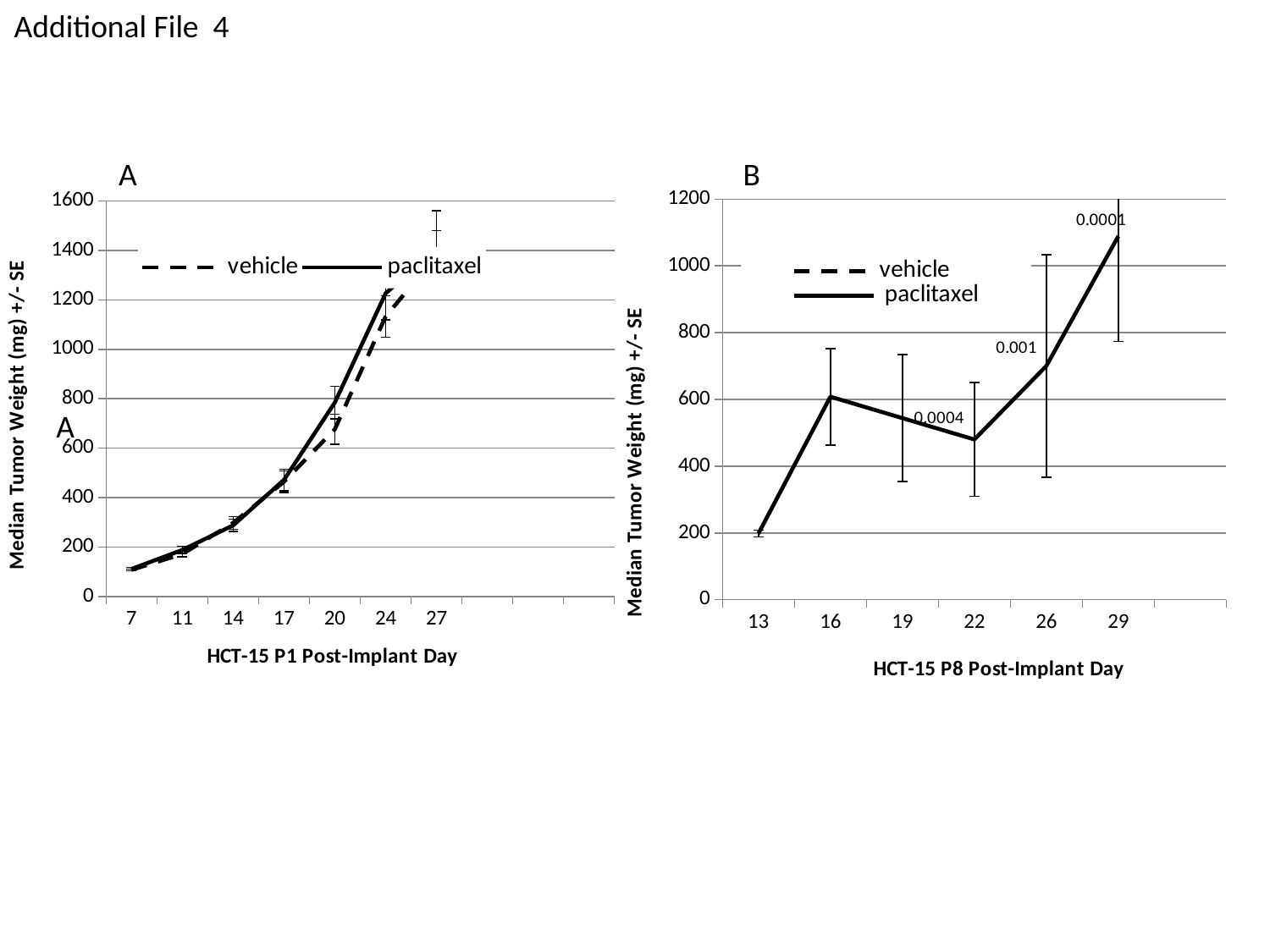

Additional File 4
A
B
[unsupported chart]
### Chart
| Category | vehicle | paclitaxel |
|---|---|---|
| 13.0 | 216.0 | 198.0 |
| 16.0 | 558.5 | 608.0 |
| 19.0 | 952.0 | 544.0 |
| 22.0 | 1188.0 | 480.0 |
| 26.0 | 1689.0 | 700.0 |
| 29.0 | 2702.0 | 1089.0 |A

Supplement: Supplementary file 4 — Additional file 4: Antitumor efficacy of paclitaxel against HCT-15 xenografts at passages 1 (P1) and 8 (P8). Vehicle treated mice received 12.5% cremaphor/12.5% ethanol/75% saline QDx5 IV while paclitaxel was administered at 10 mg/kg QDx5 IV. Statistically significant differences between the treated and control mice were determined with Student’s t-test, those points with significant paclitaxel responses are designated by showing the p value adjacent to the data point. A) P1 tumors B) P8 tumors. (PPTX 112 KB) [file 12864_2013_6082_MOESM4_ESM.pptx]
