# Supplementary material for: Gene expression profiling of 49 human tumor xenografts from in vitro culture through multiple in vivo passages - strategies for data mining in support of therapeutic studies
Source: BMC Genomics. 2014 May 22;15(1):393. doi: 10.1186/1471-2164-15-393 (PMC4041995; doi:10.1186/1471-2164-15-393)
Supplement: Supplementary file 5 — Additional file 5: Antitumor efficacy of cisplatin against MOLT-4 and A549 xenografts. Vehicle treated mice received 0.9% saline once every 4 days for 3 treatments (Q4Dx3) by the intraperitoneal (IP) route. Statistically significant differences between the treated and control mice were determined with Student’s t-test, those points with significant cisplatin responses are designated by with the p value adjacent to the data point A) MOLT-4 xenografts, the cisplatin dose was 3.24 mg/kg. B) A549 xenografts, the cisplatin dose was 6.7 mg/kg. (PPTX 108 KB) [file 12864_2013_6082_MOESM5_ESM.pptx]

## Slide 1
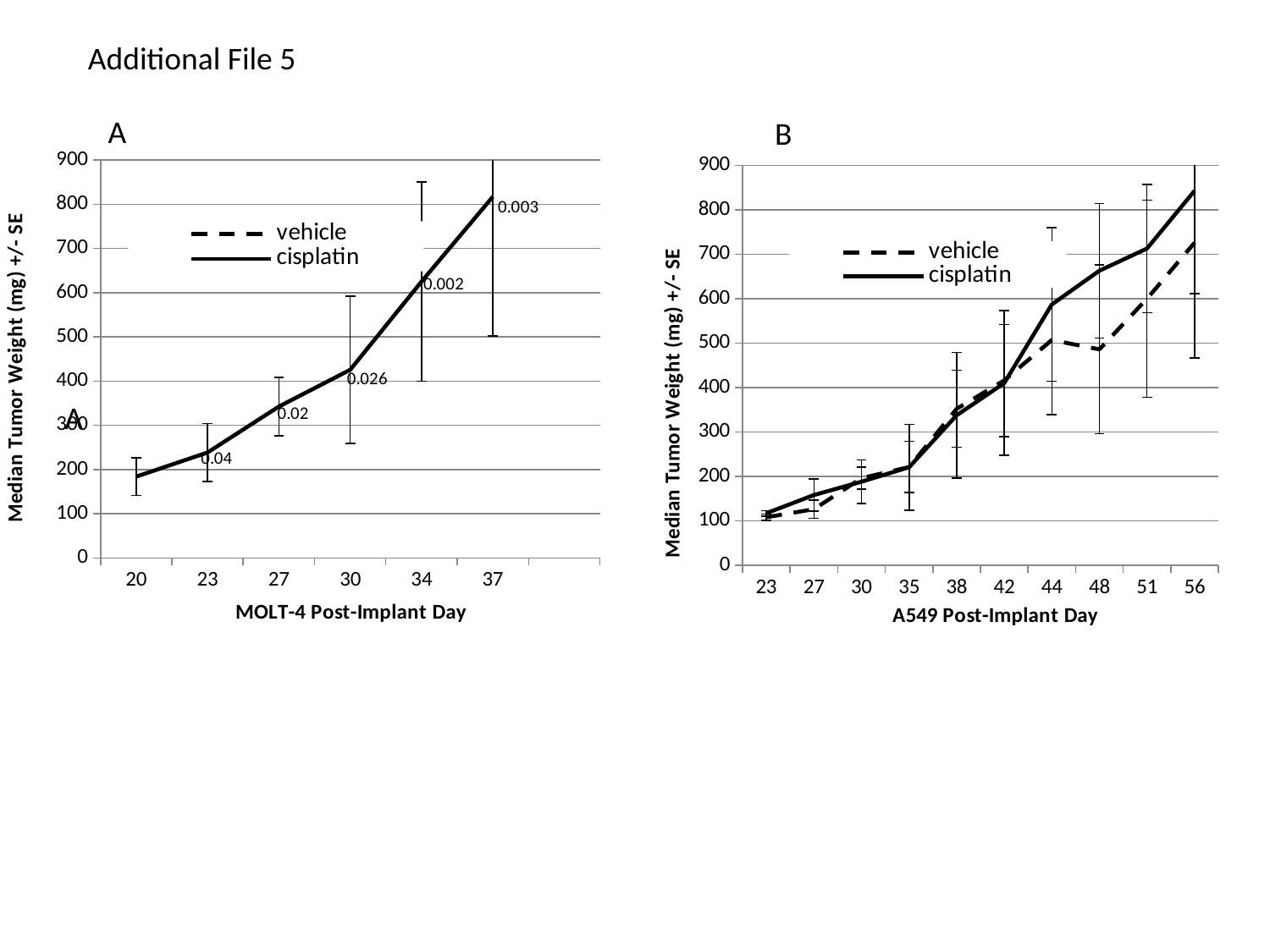

Additional File 5
A
B
### Chart
| Category | vehicle | cisplatin |
|---|---|---|
| 23.0 | 108.0 | 117.0 |
| 27.0 | 126.0 | 158.0 |
| 30.0 | 196.0 | 188.0 |
| 35.0 | 221.0 | 220.5 |
| 38.0 | 352.0 | 337.5 |
| 42.0 | 416.0 | 410.0 |
| 44.0 | 507.0 | 587.0 |
| 48.0 | 486.0 | 663.0 |
| 51.0 | 600.0 | 713.0 |
| 56.0 | 726.0 | 843.0 |
### Chart
| Category | vehicle | cisplatin |
|---|---|---|
| 20.0 | 167.0 | 184.0 |
| 23.0 | 362.5 | 238.5 |
| 27.0 | 605.0 | 342.5 |
| 30.0 | 994.0 | 425.5 |
| 34.0 | 1773.0 | 625.0 |
| 37.0 | 2461.5 | 817.0 |A
